# In Vitro Digestibility and Models of Cumulative Gas Production of Forage-Free Diet

**DOI:** 10.3390/ani13223515

**Published:** 2023-11-14

**Authors:** Luís Carlos Vinhas Ítavo, Antonio Leandro Chaves Gurgel, Camila Celeste Brandão Ferreira Ítavo, Camila Soares Cunha, Vanessa Zirondi Longhini, Gelson dos Santos Difante, Alexandre Menezes Dias, Juliana Caroline Santos Santana, Angelo Herbet Moreira Arcanjo, Marcus Vinicius Garcia Niwa, Lucimara Modesto Nonato, Geraldo Tadeu dos Santos, Alfonso Juventino Chay-Canul

**Affiliations:** 1 College of Veterinary Medicine and Animal Science, Federal University of Mato Grosso do Sul, Campo Grande 79070-900, Brazil; antonio.gurgel@ufpi.edu.br (A.L.C.G.); camila.itavo@ufms.br (C.C.B.F.Í.); camila.cunha@ufms.br (C.S.C.); vanessa.longhini@ufms.br (V.Z.L.); gelson.difante@ufms.br (G.d.S.D.); alexandre.menezes@ufms.br (A.M.D.); jukrol_@hotmail.com (J.C.S.S.); angelohmarcanjo@gmail.com (A.H.M.A.); marcusniwa@gmail.com (M.V.G.N.); lucimara.nonato@ufms.br (L.M.N.); gtsantos50@gmail.com (G.T.d.S.); 2 División Académica de Ciencias Agropecuarias, Universidad Juárez Autónoma de Tabasco, Villahermosa 86025, Mexico; aljuch@hotmail.com

**Keywords:** cumulative gas production, digestibility, effective fiber, mathematical models, nonlinear models

## Abstract

**Simple Summary:**

Diets for confined cattle are generally high in concentrated ingredients, which need attention to avoid ruminal disorders. Adding an adequate fiber quantity can help in this scenario, and by-products derived from the agroindustry can be used as a fiber source. Thus, in this research, we performed an in vitro evaluation to understand if cottonseed cake would be able to replace corn silage in a forage-free diet. In addition, the in vitro trials for feed digestion needed equations to describe the results, and it is of major importance to know if the chosen equation can adequately describe the digestion process. In the present study, it was shown that the forage-free diet containing cottonseed cake as a fiber source stimulated higher gas production and that Brody and Ørskov models presented higher precision and accuracy in explaining in vitro digestion in both diets.

**Abstract:**

Our objectives were to evaluate the use of cottonseed cake in replacing corn silage in a diet without forage and to identify the model with higher precision and accuracy of adjustment of parameters of ruminal degradation kinetics. A diet containing corn silage and another with cottonseed cake as a fiber source were formulated. Gompertz, Dual-pool Logistic, Brody, and Ørskov models were evaluated for goodness of fit to gas production. There were significant differences in dry matter (DM), organic matter (OM), and neutral detergent fiber (NDF) in the in vitro digestibility for diets and fiber sources. The estimated values of the Gompertz (6.77), Brody (6.72), and Ørskov (6.73) models were similar to the observed mean of gas production in the corn silage diet (6.73 mL/100 mg DM). Similarly, the estimated values of the Brody (5.87) and Ørskov (5.89) models were similar to the observed mean of gas production in the cottonseed cake diet (5.87 mL/100 mg DM). The roughage-free diet containing cottonseed cake as a fiber source stimulated higher gas production. Brody and Ørskov models presented higher precision and accuracy in the fitting of kinetics of degradation independent of the fiber source in the diet.

## 1. Introduction

Concentrate feeds are used in confinement diets to meet nutritional needs, improving feeding efficiency [1]. Higher concentrate:forage ratios alter the parameters of rumen fermentation, creating an unfavorable ruminal environment [2]. According to Mertens [3], the physically effective fiber, which is defined as the fraction of NDF that stimulates rumination, stimulating saliva secretion and ruminal pH buffering, can provide a suitable ruminal environment for the fermentation and degradation of nutrients, consequently providing a better performance. For finishing cattle, the NASEM [4] cites 9% as the minimum recommendation for physically effective NDF.

By-products from the agroindustry that are fiber-rich can replace forage in ruminant diets [5]. Researchers have studied cottonseed cake as an alternative to corn silage, and it has been shown to be sustainable and economically justified to provide an effective amount of fiber for feedlot beef cattle [6,7,8,9]. However, diets without roughage can lead to ruminal troubles and alterations in the metabolism of bacteria, which can compromise digestibility and animal performance [10,11,12]. Understanding how the fermentation process occurs allows the elaboration of diets that favor the fermentation of the ruminal microbiota and consequently the animal performance.

The in vitro cumulative gas production technique is frequently used to assess concentrate [13,14] and roughage quality [15,16,17]. By the measurement of in vitro gas accumulation, it is possible to know about the kinetics of feed digestion in rumen fluid. In addition, mathematical models are required to describe and explain in vitro gas production kinetics. Nevertheless, choosing the most appropriate model is fundamental [18]. Nonlinear models, especially the Gompertz, Brody, Orskov, and Dual-pool Logistic models, are used to fit the kinetics of in vitro gas production. The Dual-pool Logistic model [19] has been commonly used [13,14,15,16,17,20,21]. However, an important step in this evaluation process is the choice of the model to adjust the fermentation parameters according to the studied food.

Velho et al. [22] evaluated the Exponential, France, Gompertz, and Logistic mathematical models to study the corn silage kinetics of gas production in vitro using incubations for 24 and 48 h. These authors found that twenty-four-hour in vitro incubation periods do not mask treatment effects and concluded that the Gompertz model best explained corn silage kinetics in the in vitro gas production. In the work of Zornitta et al. [17], five mathematical models were tested, and Groot and Richards models presented the best fit for estimating data on corn silage gas production. Likewise, Gurgel et al. [21] found that the Groot and Richards models are applicable to express the in vitro gas production kinetics of diets with *Gliricidia sepium* hay or silage.

Thus, we studied the in vitro effects of the replacement of corn silage with cottonseed cake in the diet of ruminants as a fiber source on digestibility and cumulative gas production, and we identified the model with the highest precision and accuracy of adjustment of the parameters of the in vitro kinetics of degradation of the two diets.

## 2. Materials and Methods

The study was performed at the College of Veterinary Medicine and Animal Science of the Federal University of Mato Grosso do Sul, Campo Grande, MS, Brazil (20°26′50″ S, 54°50′21″ W, altitude 417 m). This study was carried out following the standard procedures of the Guide for the National Council for the Control of Animal Experiments. The protocol was approved by the Ethics Committee on Animal Use of Federal University of Mato Grosso do Sul (Protocol N° 1.181/2021). The study was reported in accordance with ARRIVE guidelines [23].

### 2.1. Experimental Design and Treatments

Isoproteic and isoenergetic diets with the same peNDF and NDF contents and total digestible nutrient (TDN, 760 g/kg) contents were formulated, replacing corn silage with cottonseed cake in the diet of beef cattle (Table 1). Samples of the diet were dried in a forced-air oven at 55 °C for 72 h, processed in knife Wiley mills with 1 mm screen sieves, identified, and stored until the analysis. The samples were analyzed according to the methodologies described by the AOAC [24] for the contents of DM (method 930.15), Ash (method 942.05), and CP (method 955.04); EE was analyzed with the Ankom^XT15^ Extractor (Ankom Technology, Macedon, NY, USA) according to the manufacturer’s instructions. The Tecnal TE-149 fiber analyzer (Tecnal, Piracicaba, SP, Brazil) was used to assess the NDF and ADF contents, using thermostable α-amylase [25]. The total digestible nutrients (TDN) were estimated using the formula described by Cappelle et al. [26]: TDN (%) = 910.246 − 5.71588 × NDF (g/kg).

The Penn State Particle Size technique was used to verify the peNDF, according to Lammers et al. [27], which consists of using a set of overlapping sieves that start with the upper one with a diameter of 19.0 mm, followed by the others with diameters of 8.0 mm and 4.0 mm, and below this, a fourth tray with a closed bottom. The technique was performed by placing approximately 300 g of each forage (corn silage or cottonseed cake) in the upper tray (19.0 mm) and shaking it back and forth five times for each side of the tray. Subsequently, the samples were dried in a ventilation oven at 55 °C, after which the DM content of the two fiber sources was gathered. Subsequently, the DM value of the samples retained on the sieves was multiplied by the NDF content of the respective forages. To determine the peNDF, the concentrations saved on the 19.0 and 8.0 mm sieves were added and determined by the volume concentration in the total diet.

**Table 1:** Ingredients and chemical composition of experimental diets *.

	Diet	
	Corn Silage	Cottonseed Cake
Corn silage (g/kg DM)	200	-
Cottonseed cake (g/kg DM)	-	230
Ground corn (g/kg DM)	619	740
Cottonseed meal (g/kg DM)	155	-
Urea (g/kg DM)	6	10
Supplement mineral (g/kg DM)	20	20
Chemical composition (g/kg)		
DM	658	879
OM	954	959
CP	147	151
EE	41	56
NDF	277	267
peNDF	97	94
TDN	761	761

DM: dry matter; OM: organic matter; CP: crude protein; EE: ether extract; NDF: neutral detergent fiber; peNDF: physically effective neutral detergent fiber; TDN: total digestible nutrients. * Diets formulated according to BR-Corte (2016) for average gains of 1.5 kg/day according to Valadares Filho et al., [28] using corn silage or cottonseed cake as fiber source.

### 2.2. In Vitro Digestibility

The methodologies described by Tilley and Terry [29], modified as suggested by Holden [30] to the Ankom system (Ankom Technology Corp., Macedon, NY, USA), were used for in vitro digestibility evaluation. All bags used were washed with acetone, aiming to remove surfactants, which are capable of inhibiting microbial digestion. Then, samples of all treatments were weighed into polypropylene synthetic tissue filter bags (5 × 5 cm in size with a 50 μm pore size). Six bags containing 0.5 g of sample were incubated, three for the analysis of ash and organic matter and three for the analysis of dry matter and neutral detergent fiber, in jars fitted with Bunsen valves (30 bags per flask, 6 blank filter bags for contamination correction). Jars contained 1.6 L of buffer solution (solution A = (g/L), 10.0 g of KH_2_PO_4_, 0.5 g of MgSO_4_7H_2_O, 0.5 g of NaCl, 0.1 g of CaCl_2_2H_2_O, and 0.5 g of urea; solution B = (g/100 mL), 15.0 g Na_2_CO_3_, 1.0 g Na_2_S9H_2_O). The final solution had an A:B ratio of 5:1 and a pH of 6.8. Animals whose primary diet consisted solely of grass had their rumen fluid collected; the strained rumen fluid, totaling 400 mL, was transported at a regulated temperature and put into each flask, which were purged with CO_2_ for 5 s and were incubated for 48 h at a constant temperature (39 °C) with continuous shaking. Subsequently, 40 mL of 6 N HCl and 8 g of pepsin were added to each flask. The flasks were incubated for an additional 24 h, and at the end of 72 h, the bags were washed with distilled water and then oven-dried at 105 °C for 16 h. We used blanks to estimate a correction factor that adjusted for weight changes from the sample bags [31]. One unique incubator was used to keep all jars, with the temperature fixed at 39 °C. After the incubation time, the bags were dried and weighed. A correction for bacterial contamination using blank bags [31] was performed. The in vitro digestibility of DM (ivDMD), OM (ivDOMD), and NDF (ivDNDF) was calculated from differences between the amount of nutrients in the feed and that in the residue after incubation [13]. These analyses were carried out three times to obtain repetitions of the in vitro digestibility results.

### 2.3. In Vitro Gas Production Data

In vitro gas production for the ground diet was performed as suggested by Theodorou et al. [32] and was adapted for the Ankom RF Gas Production System (Ankom Technology, NY, USA). Initially, all vials were purged with CO_2_. Then, triplicate samples (0.5 g) per treatment were added to vials (310 mL), and 100 mL of buffer solution was preheated to 39 °C, aiming for a final pH of 6.8. The vials were kept under a controlled temperature (39 °C) and agitation. The pressure (psi) of each vial was recorded every 5 min for 48 h and processed for cumulative gas production (mL gas/100 mg DM incubated). The pressure data in terms of volume were converted for cumulative gas production and corrected for blanks [17]. Three running were carried out to obtain repetitions of the in vitro gas production results.

### 2.4. Models and Curve-Fitting

In vitro gas data were fitted into four mathematical models (Table 2). The equations of the Gompertz and Dual-pool Logistic sigmoidal models are described by Schofield et al. [19]. The exponential models, Brody and Ørskov, are described by Brody [33] and Ørskov and McDonald [34], respectively.

### 2.5. Statistical Analysis

The model parameters were estimated by the modified Gauss Newton method using the SAS NLIN procedure (SAS University Edition, SAS Institute Inc. Cary, CA, USA). The maximum number of interactions used was 100. The criteria for evaluating the adequacy of the equations were as follows: coefficient of determination (R^2^); F test for the identity of the parameters (β0 = 0 and β1 = 1) of the regression predicted for observed data; concordance correlation coefficient (CCC); root mean square error of prediction (RMSEP); and decomposition of the mean square error of prediction (MSEP) into mean error, systematic bias, and random error [35], using Model Evaluation System software version 3.2.2. A significant level of 5% was adopted for all statistical analyses. 

The total in vitro gas production and digestibility were analyzed by a one-way analysis of variance using the General Linear Models procedure of the SAS statistical package (SAS University Edition, Sas Institute Inc., Cary, NC, USA). The means among the treatments were compared by the Fisher’s test at a probability of α = 0.05 using the following statistical model: Y_ij_ = µ + D_i_ + e_ij_, where: μ = general mean; D_i_ = diet effect i, with i ranging from 1 to 2 (1 = corn silage as a fiber source in the diet, and 2 = cottonseed cake as a fiber source in the diet); e_ij_ = random error associated with each observation.

## 3. Results

### 3.1. In vitro Digestibility

The in vitro digestibility of DM, OM, and NDF presented significant differences between diets (*p* < 0.001). The corn silage diet showed greater DM and NDF digestibility and smaller OM digestibility. The in vitro digestibility of DM, OM, and NDF was different (*p* < 0.001) between the fiber sources isolated. Similar to the results from the diets, the corn silage showed greater DM and NDF digestibility and reduced OM digestibility (Table 3).

### 3.2. Models and Curve-Fitting

The estimated values of the Gompertz, Brody, and Ørskov models were similar to the observed mean of the total gas production in the corn silage diet (β0 = 0 and β1 = 1). Similarly, the estimated values of the Brody and Ørskov models were similar to the observed mean of the total gas production in the cottonseed cake diet. These models showed similar results regarding the concordance correlation coefficient (CCC) and coefficient of determination (R^2^) and presented a smaller root mean square error of prediction (RMSEP) (Table 4).

The Gompertz, Brody, and Ørskov models were best able to predict the variability observed between the times of total gas production (R^2^ > 0.85) for the assessment of the corn silage diet. In the assessment of the cottonseed cake diet, the adequacy of the models tested showed that only the Brody and Ørskov models could predict the variability detected between the times of total gas production (R^2^ > 0.99), despite that the Gompertz and Dual-pool Logistic models presented high determination coefficients as well (R^2^ > 0.99).

For the corn silage diet, the concordance correlation coefficient (CCC) analysis showed that the Brody and Ørskov models showed higher accuracy and precision, with similar behavior observed in the mean square error (RMSEP). The decomposition of the mean square error of prediction (MSEP) showed that, in the Brody and Ørskov models, the observed deviations can be attributed to the random error (100%), showing no mean or systematic deficiency of the model. In the Dual-pool Logistic model, 2.674% of the deviation was linked to systematic bias, demonstrating a multiplicative error in the predicted values and a higher concentration in the mean error (96.156%), showing the overprediction of digestion by the model for the cottonseed cake diet. The concordance correlation coefficient (CCC) analysis showed that the Brody and Ørskov models showed higher accuracy and precision, with similar behavior observed in the mean square error (RMSEP, 0.41). The decomposition of the mean square error of prediction (MSEP) showed that, in the Brody and Ørskov models, the observed deviations can be attributed to the random error (99.657 and 99.992%, respectively), showing no mean or systematic deficiency of the model. In the Gompertz and Dual-pool Logistic models, 34.754 and 9.688% of the deviation were associated with systematic bias, respectively, demonstrating a multiplicative error in the predicted values and a higher concentration in the mean error, showing an over-prediction of the model (Table 4).

### 3.3. In Vitro Gas Production Data

**Figure 1:**
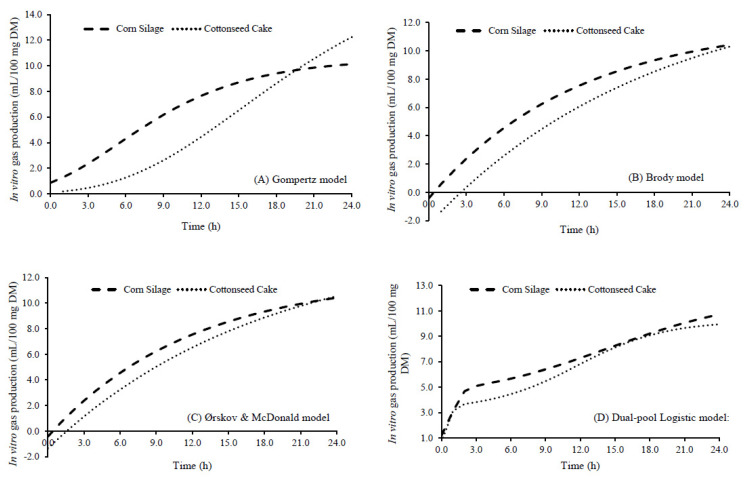
Modeling of total gas production estimated by the models of the in vitro gas production of diets containing corn silage or cotton cake as a fiber source. (**A**) Gompertz model: Y_corn silage_ = 10.55 × e^(−2.50×e(0.17t))^; Y_cottonseed cake_ = 18.09 × e^(−4.53×e(0.1t))^. (**B**) Brody model: Y_corn silage_ = 12.05(1 − 1.04 × e^(0.08t)^); Y_cottonseed cake_ = 14.72(1 − 1.09 × e^(0.06t)^). (**C**) Ørskov and McDonald model: Y_corn silage_ = −0.43 +12.48 × (1 − e^(−0.08t)^); Y_cottonseed cake_ = −1.36 + 16.07(1 − e^(−0.06t)^). (**D**) Dual-pool Logistic model: Y_corn silage_ = 4.04/(1 + e^(2−4×0.26(t*−*2.19))^) + 8.16/(1 + e^(2−4×0.04(t−2.19))^); Y_cottonseed cake_ = 3.18/(1 + e^(2−4×0.23(t−4.19))^) + 7.02/(1 + e^(2−4×7.02(t−4.19))^).

The modeling of total gas production was estimated by the models of the in vitro gas production of diets containing corn silage or cottonseed cake as a fiber source and showed differences in the fitted parameters of the models (Figure 1). The Gompertz model fitted the following models to the corn silage diet Y_corn silage_ = 10.55 × e^(−2.50×e(0.17t))^ and to the cottonseed cake diet Y_cottonseed cake_ = 18.09 × e^(−4.53.e(0.1t))^. The Brody model presented the adjustment as follows: Y_corn silage_ = 12.05(1 − 1.04 × e^(0.08t)^), and Y_cottonseed cake_ = 14.72(1 − 1.09 × e^(0.06t)^). The Ørskov and McDonald model was fitted to the corn silage diet Y_corn silage_ = −0.43 + 12.48(1 − e^(−0.08t)^) and to the cottonseed cake diet Y_cottonseed cake_ = −1.36 + 16.07(1 − e^(−0.06t)^). The Dual-pool Logistic model showed the following equations: Y_corn silage_ = 4.04/(1 + e^(2−4 × 0.26(t−2.19))^) and Y_cottonseed cake_ = 3.18/(1 + e^(2−4 × 0.23(t−4.19))^) + 7.02/(1 + e^(2−4 × 7.02(t−4.19))^). It should be noted that the parameters of the adjusted models were different from each other for the same diets and for the different models.

**Figure 2:**
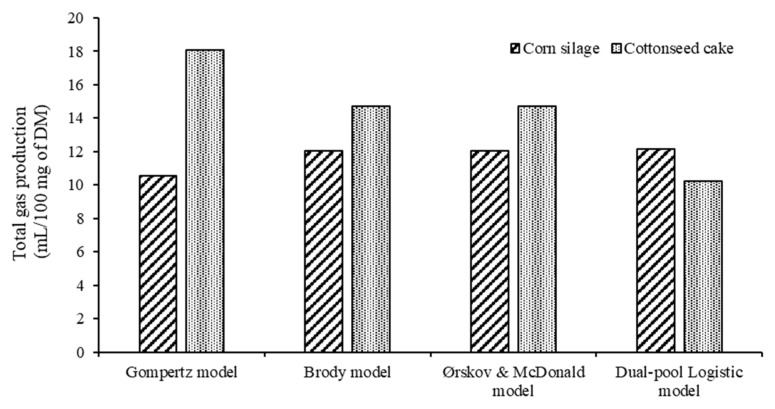
Total gas production estimated by the models of in vitro gas production of diets containing corn silage or cottonseed cake as a fiber source.

There were no significant differences in total gas production (mL of gas/100 mg DM) for the Brody (12.05), Ørskov (12.05), and Dual-pool Logistic (12.17) models in the corn silage diet. While, for the cottonseed cake diet, only the Brody (14.72) and Ørskov (14.71) models presented similarity in total gas production (Figure 2).

## 4. Discussion

### 4.1. In Vitro Digestibility

When used as the only source of fiber in diets lacking in roughage, cottonseed cake can replace the fiber from corn silage for cattle in feedlots, which favors the operational management of confinements and reduces production costs [9]. However, our study tested the effects of the replacement of cottonseed cake by corn silage in the diet of ruminants as a fiber source on the digestibility and kinetics of fermentation. We observed that fiber sources altered the in vitro digestibility, with greater results for corn silage. Despite maintaining the same NDF content of the diet, cottonseed fiber was less effective in maintaining rumination activity. Biologically, forages are fiber-rich foods that stimulate and require mastication, influencing the rate of passage and the biphasic nature of the rumen [36]. Negrão et al. [37] warned that the use of cottonseed cake in sheep diets needs attention since this ingredient can lead to linear reduction in nutrient voluntary intake and digestibility. Moreover, the inclusion of cottonseed cake can change the quantities of digestible and indigestible fractions in the diet.

Furthermore, the laboratory methods [4,27] are not standardized to measure particle size and estimate the effectiveness of fiber from agro-industry co-products, in addition to providing weak associations with rumen parameters in high-concentrate diets [6]. Diets can alter almost 50% of rumen flora bacteria species [38]. Thus, these variations observed in the choice of model in our results can be attributed to ruminal flora differences between diets. Negrão et al. [39] found that cottonseed cake in sheep diets replacing soybean meal did not significantly modify the blood parameters and led to slight variations in the ruminal parameters, mainly in the ruminal ammonia–nitrogen without affecting the sheep’s health. They highlighted that further research is necessary to explain the effect of cottonseed cake on animal health in other categories.

Arcanjo et al. [8] observed lower in vitro digestibility of DM and NDF in the cottonseed cake fractions that passed through the 8 mm sieve in the Penn State Particle Size evaluation, in relation to the corn silage samples. In addition, the authors observed that cattle fed a diet with cottonseed cake had lower rumination time and bite rate. According to Zhou et al. [40], particles ≤ 1.18 mm may have lower digestibility because they have a higher rumen passage rate, mainly affecting fiber digestibility due to the low microbial action.

### 4.2. Models and Curve-Fitting

Statistical techniques used to evaluate the precision and accuracy of the models are countless. However, no technique used in isolation is capable of adequately evaluating the performance of the models [35]. In our work, initially, the models were evaluated by the F test for the identity of the parameters (β0 = 0 e β1 = 1) of the regression of predicted observed data. Based on this test, the Dual-pool Logistic model (β0 ≠ 0 and β1 ≠ 1), independent of the fiber source, and the Gompertz model for the cottonseed cake diet estimated values different from the observed. The CCC and R^2^ of the Brody and Ørskov models showed larger accuracy and precision to estimate the parameters of kinetic fermentation. Furthermore, most errors can be attributed to the aleatory component. Thus, Brody and Ørskov models would be more adequate to explain the kinetic fermentation of the diets containing distinguished fiber sources.

Velho et al. [22] recommended the Gompertz model to adjust the kinetics of in vitro gas production in corn silages. The Groot model had the best fit for estimates of the in vitro gas production data of corn silage [17]. These divergences regarding the different adjusted models are understandable, once the fitting depends on the intrinsic characteristics of the food under study [21]. Cottonseed cake has a greater lipid content and fermentation can be reduced with the increase in its inclusion, in addition to making it difficult for bacteria to adhere to food particles [41]. Thus, it is necessary to evaluate the appropriate model for each diet situation [42].

### 4.3. In Vitro Gas Production Data

The volume of gas production was affected by the fiber source; however, ruminal kinetics is a reflection of the microorganisms on the components of the diet, digestibility, and ruminal flora to start the colonization. The inclusion of cottonseed cake in the diet increases the fat content that reaches the rumen, and this element can impair fermentation, making it difficult for rumen bacteria, especially cellulolytic, to access the food particle. Wanderley et al. [43] observed a reduction in total gas production when cottonseed was included in dairy cows’ diets. This fact may be related to the change in ruminal bacterial diversity, since the diet is able to stimulate specific groups of bacteria [38].

The lag time estimated by the Dual-pool Logistic model of the cottonseed cake diet presented 4.19 h, and the lag time of the corn silage diet was 2.19 h (Figure 1). The different fiber sources affected the colonization time, due to its chemical composition, such as the type of fiber that constitutes the material to be degraded by ruminal microorganisms, reaffirming that the ability to use cottonseed cake in the total diet should be carried out with caution [37,39]. Silva et al. [41] also observed a longer colonization time of ruminal bacteria, but with stabilization between diets with cottonseed cake. Despite that, Arcanjo et al. [9] evaluated the productive performance of Nellore steers finished in feedlots using 300 g/kg of cottonseed cake in the total diet as the only source of fiber and observed the positive effect of cottonseed cake as the only dietary fiber source for final body weight, total weight gain, hot carcass weight, and carcass yield.

## 5. Conclusions

A forage-free diet containing cottonseed cake as a fiber source stimulated higher gas production.

Independent of the fiber source, in vitro fermentation kinetic parameters in ruminant forage-free diets were better adjusted by the models of Brody and Ørskov, which were more adequate and presented higher precision and accuracy.

## Figures and Tables

**Table 2:** Nonlinear models considered in this study to describe the in vitro gas production of diets containing corn silage or cottonseed cake as a fiber source.

Models	Equation	Parameters
Gompertz	*V*(*t*) *= V_F_* e^(−*b.*e(*−kt*))^	3
Brody	*V*(*t*) *= V_F_* (1 − *b.*e^(^*^−kt^*^)^)	3
Ørskov and McDonald	*V*(*t*) *= V_F_ + b*(1 *−* e^(^*^−kt^*^)^)	3
Dual-pool Logistic	*V*(*t*) *= V*_1*F*_/(1 + e^(2−4.*k*1(*t−*^*^λ^*^))^) + *V*_2*F*_/(1 + e^(2−4.^*^k^*^2(*t−*^*^λ^*^))^)	5

*V(t)* is the cumulative gas production (mL/100 mg of DM) in time *t*, *V_F_* is the maximum potential of gas production (mL/100 mg of DM), *V_1F_* is the final volume of gases derived from the degradation of the rapid digestion soluble fraction (fractions A+ B1 of the Cornell System) when T→∞, *V_2F_* is the final volume of gases derived from the degradation of the slow digestion insoluble fraction (fraction B2 of the Cornell system) when T→∞, *k* represents the fractional rate of gas production (%/h), *k*_1_ is the specific gas production rate due to the degradation of the soluble fraction (%/h), *k*_2_ is the specific gas production rate due to the degradation of the insoluble fraction (%/h), *t* is the incubation time (*h*), λ is the lag time (*h*), b represents the interaction constant, and *e* is exponential.

**Table 3:** The in vitro digestibility (g/kg) of the diets and the fiber sources.

	Diets		SEM	*p*-Value
	Corn Silage	Cottonseed Cake		
*iv*DDM	958.0 a	935.4 b	9.95	0.0001
*iv*DOM	875.2 b	883.2 a	3.20	0.0001
*iv*DNDF	871.4 a	846.6 b	9.21	0.0001
	Fiber source			
	Corn silage	Cottonseed cake		
*iv*DDM	834.7	570.9	6.68	0.0001
*iv*DOM	882.1	940.4	0.66	0.0001
*iv*DNDF	765.2	538.5	3.68	0.0001

*iv*DDM = in vitro digestibility of dry matter; *iv*DNDF = in vitro digestibility of neutral detergent fiber; *iv*DOM = in vitro digestibility of organic matter. SEM: standard error of mean. a,b: Mean within a row with different letters differ significantly at *p* < 0.05.

**Table 4:** Evaluation of the equations developed to describe the in vitro gas production of diets containing corn silage or cottonseed cake as fiber source.

	Mean	SD	Min	Max	R^2^	*p*-Value	CCC	RMSEP	Decomposition of MSEP (%)		
									ME	SB	RE
Corn silage diet											
Observed data	6.73	3.62	0.0	13.57							
Gompertz	6.77	2.96	0.86	10.13	0.84	0.7978	0.82	1.95	0.038	0.194	99.767
Brody	6.72	3.08	−0.43	10.42	0.85	0.9999	0.84	1.91	0.000	0.000	100.00
Ørskov and McDonald	6.73	3.08	−0.43	10.42	0.85	0.9999	0.84	1.91	0.000	0.000	100.00
Dual-pool Logistic	6.94	2.77	0.85	10.71	0.85	0.0220	0.82	1.92	1.170	2.674	96.156
Cottonseed cake diet											
Observed data	5.87	3.47	0.0	10.09							
Gompertz	5.59	4.09	0.19	12.72	0.95	0.0001	0.94	1.33	4.503	34.754	60.743
Brody	5.87	3.44	−1.37	10.54	0.99	0.9957	0.99	0.41	0.009	0.334	99.657
Ørskov and McDonald	5.89	3.44	−1.36	10.54	0.99	0.9963	0.99	0.41	0.007	0.001	99.992
Dual-pool Logistic	5.90	3.41	0.31	9.94	0.99	0.0015	0.99	0.16	3.063	9.688	87.249

SD = standard deviation; R² = coefficient of determination; *p*-value = probability value associated with the simultaneous F-test for the identity of parameters (β0 = 0 and β1 = 1) of the regression of observed vs. predicted data; CCC = concordance correlation coefficient; RMSEP = root mean square error of prediction; MSEP = mean square error of prediction. ME = mean error; SB = systematic bias; RE = random error.

## Data Availability

Data that support the study findings are available from the corresponding author upon reasonable request.
